# Serial changes in pleural lavage cytology during lung cancer surgery predict recurrence and pleural dissemination

**DOI:** 10.1007/s00595-025-03159-y

**Published:** 2025-10-17

**Authors:** Go Kamimura, Masaya Aoki, Satomi Imamura, Shoichiro Morizono, Yuto Nonaka, Takuya Tokunaga, Aya Harada-Takeda, Koki Maeda, Toshiyuki Nagata, Kazuhiro Ueda

**Affiliations:** Department of General Thoracic Surgery, Graduate School of Medical and Dental Sciences, Kagoshima University, 8-35-1 Sakuragaoka, Kagoshima, 890-8520 Japan

**Keywords:** Pleural lavage cytology, Pleural lavage cytology immediately after thoracotomy, Pleural lavage cytology at chest closure

## Abstract

**Purpose:**

Pleural lavage cytology (PLC) is a recognized prognostic marker in non-small cell lung cancer (NSCLC); however, the impact of serial intraoperative changes remains unclear.

**Methods:**

We retrospectively analyzed 439 patients who underwent curative NSCLC resection. PLC was performed at three intraoperative points: after thoracotomy (pre-PLC), after lung resection, and after lavage at chest closure (post-PLC). Associations between recurrence-free survival (RFS) and pleural dissemination were evaluated by a Kaplan–Meier analysis and Fine and Gray competing risks regression.

**Results:**

Forty-one patients had at least one positive PLC result. RFS was the lowest in pre-PLC( +)/post-PLC( +) (*n* = 10), intermediate in pre-PLC(–)/post-PLC( +) (*n* = 11), and best in post-PLC( −) (*n* = 20). Importantly, post-PLC( −) patients included 13 patients with pre-PLC positivity, yet their RFS matched that of consistently negative cases (*n* = 398). The cumulative incidence of pleural dissemination exhibited a similar pattern. In a multivariate analysis, post-PLC positivity, but not pre-PLC positivity, independently predicted poor RFS (hazard ratio, 3.06; *p* < 0.001).

**Conclusion:**

Post-PLC, but not pre-PLC, provides decisive prognostic information for recurrence and pleural dissemination, likely reflecting residual lavage-resistant tumor clusters. Importantly, combining pre- and post-PLC results refines risk stratification and identifies the poorest-outcome subgroup that may benefit from adjuvant therapy.

## Introduction

Lung cancer remains the leading cause of cancer-related mortality worldwide, with non-small cell lung cancer (NSCLC) accounting for approximately 85% of all cases and contributing substantially to cancer-related deaths [1]. Even after complete surgical resection, recurrence occurs in up to 30–50% of patients 2, underscoring the need for additional prognostic markers to refine risk stratification and guide the selection of patients who may benefit from intensified adjuvant therapy.

PLC is an established independent prognostic marker for NSCLC [3]; however, it is not incorporated into the current tumor-node-metastasis (TNM) staging system [4, 5]. Unlike malignant pleural effusion, PLC positivity did not alter tumor stage. In most institutions, PLC is performed only once, typically immediately after thoracotomy (Pre-PLC) [6], whereas only a few centers obtain additional samples during chest closure (Post-PLC).

Enatsu et al. evaluated cytology both at thoracotomy and chest closure in a large cohort, demonstrating that positivity at chest closure independently predicted poor survival. However, previous studies have not investigated how cytology results change across multiple intraoperative time points. At our institution, PLC specimens were collected at three standardized intervals: immediately after thoracotomy, after lung resection, and after a lavage (using 3 L of distilled water) of the pleural cavity at chest closure. Distilled water was used to exploit its osmotic cytotoxic effect against residual tumor cells.

In this study, we examined the changes in PLC results across these three sampling points and evaluated their association with postoperative recurrence and survival in patients undergoing curative resection for NSCLC. Clarifying the prognostic impact of these transitions may help refine risk stratification and inform postoperative management, including the selection of patients who are most likely to benefit from adjuvant therapy.

## Materials and methods

Between June 2017 and December 2021, 580 patients with primary NSCLC underwent curative resection at our institution. Surgical procedures ranged from sublobar resection (wedge resection or segmentectomy) to pneumonectomy, with lobectomy being performed in the majority of cases. Systematic or selective lymph node dissection was performed at the discretion of the surgeon. After excluding patients with inadequate cytology submission, incomplete resection, disseminated disease, or insufficient follow-up, 439 patients were included in the analysis.

PLC was performed at three standardized intraoperative time points: immediately after thoracotomy (PLC1), after lung resection (PLC2), and at chest closure after pleural lavage (PLC3). At each time point, 100 mL of normal saline was instilled into the pleural cavity and 50 mL was retrieved for cytological examination. For PLC3, the pleural cavity was first irrigated with 3000 mL of distilled water to induce osmotic cytotoxicity, after which lavage with 100 mL of saline and retrieval of 50 mL was performed. Among the 41 patients with at least one positive PLC result, there were 70 instances of positivity across all sampling points, including 8 judged as suspicious. All suspicious samples were considered positive for the purpose of this study.

Pathological staging was determined according to the 7th edition of the TNM classification [8]. Pleural invasion (PL) was defined as tumor infiltration beyond the elastic layer of the visceral pleura, and lymphovascular invasion (LVI) was considered positive if either vascular or lymphatic invasion was identified. Follow-up examinations were performed every 3–6 months and included physical assessments, chest computed tomography, and positron emission tomography/computed tomography, when indicated.

### Statistical analyses

Categorical variables were compared using the Fisher’s exact test. Recurrence-free survival (RFS) was estimated using the Kaplan–Meier method and compared using the log-rank test, and pairwise P-values were Bonferroni-adjusted for three-group comparisons. The cumulative incidence of pleural dissemination and malignant pleural effusion was estimated using the cumulative incidence function and compared using Gray’s test with Bonferroni adjustment for pairwise comparisons. To evaluate the impact of PLC after thoracotomy and PLC at chest closure on recurrence and lung cancer-specific death, the primary event was defined as either recurrence or lung cancer death, with deaths due to other causes considered as competing events. To identify independent predictors of RFS while accounting for non-lung cancer death as a competing event, we performed Fine and Gray competing risks regression, initially in univariate analyses and subsequently in a multivariable model that included four clinically essential variables without additional confounders, owing to the limited number of events. Overall, 69 RFS primary events and 23 competing (non-lung cancer) deaths occurred during the follow-up period. The median follow-up duration was estimated using the reverse Kaplan–Meier method by reversing the event indicator (deaths treated as censored and censoring treated as events). The median follow-up was 24.5 months (95% CI 22.8–26.8). Statistical significance was set at *p* < 0.05. All statistical analyses were conducted using the EZR software program (Jichi Medical University, Japan).

## Results

**Fig. 1:**
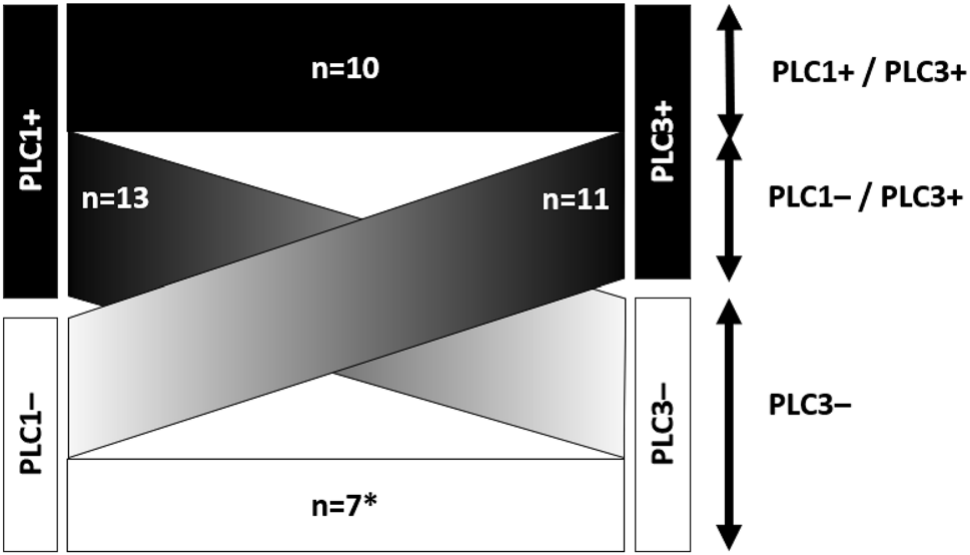
Transition of serial PLC among any-positive cases (*n* = 41). Patients with at least one positive PLC were stratified by the results immediately after thoracotomy (PLC1) and at chest closure (PLC3) to depict transition across surgery. Four patterns were observed: persistently positive (PLC1 + /PLC3 +), conversion-to-positive (PLC1–/PLC3 +), conversion-to-negative (PLC1 + /PLC3–), and PLC2-only positive (PLC1–/PLC3– with PLC2 +). The asterisk denotes cases that were positive only after lung resection (PLC2 +), while PLC1 and PLC3 were negative

Table 1 summarizes the distribution of the PLC1–3 results, which were categorized into eight transition patterns. Of the 439 patients, 398 remained negative at all three time points (consistently negative group), whereas 41 showed positivity at one or more points. Among these 41 patients, 20 were negative at PLC3 and 21 were positive at PLC3, either persistently positive (PLC1 + / PLC3 +) or converting from negative to positive (PLC1– / PLC3 +) (Fig. 1).

**Table 1:** Serial cytology results at three timepoints (n = 439)

Types		Serial PLC		
	PLC1	PLC2	PLC3	n
Type 1	Positive	Positive	Positive	7
Type 2	Positive	Positive	Negative	5
Type 3	Positive	Negative	Positive	3
Type 4	Positive	Negative	Negative	8
Type 5	Negative	Positive	Positive	7
Type 6	Negative	Positive	Negative	7
Type 7	Negative	Negative	Positive	4
Type 8	Negative	Negative	Negative	398

*PLC* pleural lavage cytology

Table 2 compares the clinicopathological characteristics between consistently negative patients and those with positive PLC. The median age was 70 years, 233 patients (53%) were male, and 199 (45%) were smokers. Smoking was significantly more frequent in the any-positive group (*p* = 0.006). Surgical procedures included lobectomy (*n* = 347), wedge resection (*n* = 44), segmentectomy (*n* = 41), and pneumonectomy (*n* = 7) with lymph node dissection in 314 patients (72%). No significant surgical differences were observed between the two groups. Histologically, adenocarcinoma accounted for 380 cases (86%), squamous cell carcinoma for 52 (12%), and others for 7 (2%). Pathological nodal involvement was found in 120 patients (27%), LVI in 144 patients (33%), and PL in 88 patients (20%). Only PL was significantly more frequent in the any-positive group (*p* < 0.001).

**Table 2:** Clinical characteristics of the consistently negative PLC group and the any-positive PLC group

Factors	Consistently negative PLC *n* = 398	Any-positive PLC *n* = 41	*p*-value
Sex			0.685
Female	188 (47%)	18 (44%)	
Male	210 (53%)	23 (56%)	
Age Mean (range)	70 (30–90)	71 (41–90)	0.205
Smoking			
Ever	172 (43%)	27 (66%)	0.006
Never	226 (57%)	14 (34%)	
Surgical procedure			0.235
Wedge resection	41 (10%)	3 (7%)	
Segmentectomy	41 (10%)	0 (0%)	
Lobectomy	311 (78%)	36 (88%)	
Pneumonectomy	5 (2%)	2 (5%)	
Lymph node dissection			
Yes	288 (72%)	26 (63%)	0.275
No	110 (28%)	15 (37%)	
Histology			0.089
Adenocarcinoma	348 (87%)	32 (78%)	
Squamous cell carcinoma	45 (11%)	7 (17%)	
Others	5(2%)	2 (5%)	
Pathological nodal involvement			
Present	106 (27%)	14 (34%)	0.357
Absent	292 (73%)	27 (66%)	
Lymphovascular invasion			
Present	136 (34%)	18 (44%)	0.215
Absent	262 (66%)	23 (56%)	
Pathological pleural invasion			
Present	68 (17%)	20 (49%)	< 0.001
Absent	330 (83%)	21 (51%)	

**Fig. 2:**
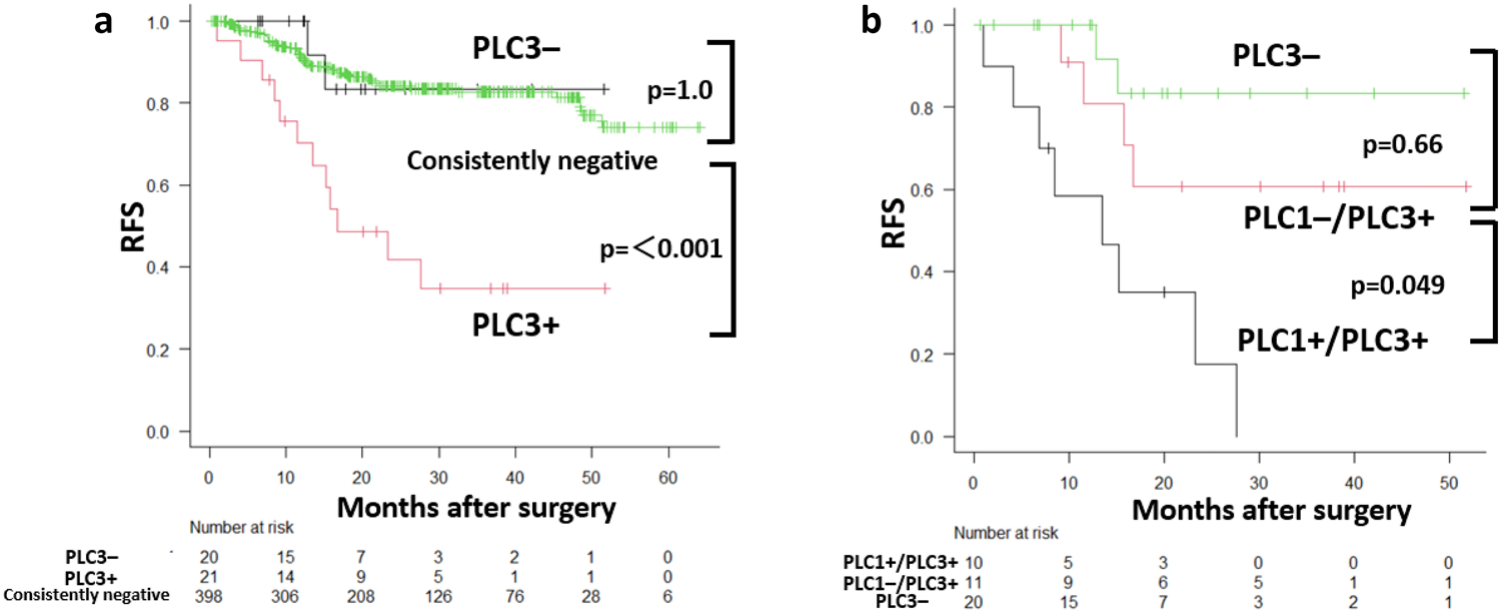
RFS according to PLC transition. **a** Kaplan–Meier RFS curves comparing consistently negative, closure-negative (PLC3–), and closure-positive (PLC3 +) groups. Pairwise log-rank p-values with Bonferroni adjustment were annotated (e.g., consistently negative vs. PLC3 + : *p* < 0.001; closure-negative vs. PLC3 + : *p* = 0.049; consistently negative vs. PLC–: *p* = 1.0). **b** RFS in any-positive PLC patients (*n* = 41) stratified by the PLC1/PLC3 combinations. Patients with at least one positive PLC were grouped by the result of PLC1 and PLC3 into three categories: post-closure negative (PLC3–), persistently positive (PLC1 + /PLC3 +), and conversion-to-positive (PLC1–/PLC3 +). Pairwise log-rank p-values with Bonferroni adjustment were annotated (e.g., PLC3 vs. PLC1–/PLC3 + : *p* = 0.66; PLC1–/PLC3 + vs. PLC1 + /PLC3 + : *p* = 0.049)

The median follow-up was 24.5 months (95% CI 22.8–26.8). Overall, 69 RFS primary events and 23 competing (non-lung cancer) deaths occurred during the follow-up period. The analysis of RFS demonstrated that patients with PLC3 + had significantly higher recurrence rates than consistently negative cases (*p* < 0.001), whereas patients with PLC3– showed outcomes similar to consistently negative patients (*p* = 1.000) (Fig. 2a). Among patients with ≥ 1 positive PLC, we stratified the outcomes by the PLC1/PLC3 combination into three groups: post-closure negative (PLC3 −), persistently positive (PLC1 + /PLC3 +), and conversion-to-positive (PLC1 − /PLC3 +). There was no significant difference between PLC3 − and PLC1 − /PLC3 + (p = 0.66), whereas the persistently positive group showed worse RFS than the conversion-to-positive group (PLC1 − /PLC3 + vs. PLC1 + /PLC3 + : *p* = 0.049) (Fig. 2b).

**Fig. 3:**
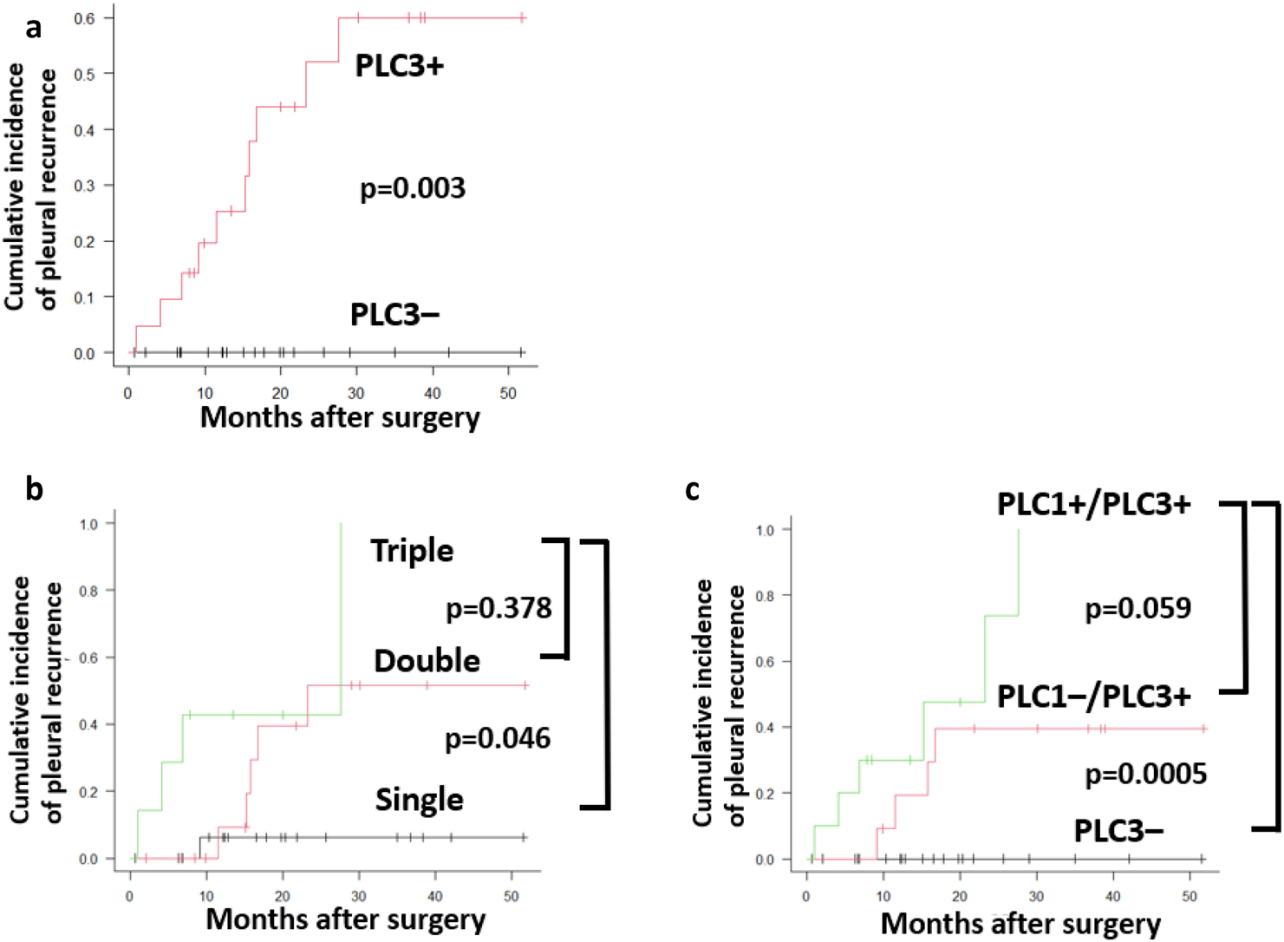
Cumulative incidence of pleural dissemination and malignant effusion. **a** Cumulative-incidence curves according to PLC3 status PLC3– vs. PLC3 + (*p* = 0.003). **b** Cumulative incidence according to the number of positive cytology results (Single, Double, Triple) Bonferroni-adjusted pairwise Gray’s tests: single vs. triple *p* = 0.046, double vs. triple *p* = 0.378. **c** Cumulative incidence by specific PLC1–PLC3 combinations. Bonferroni-adjusted pairwise Gray’s tests: PLC3 − vs. persistently positive (PLC1 + /PLC3 +) *p* = 0.0005; PLC3 vs. conversion-to-positive (PLC1 − /PLC3 +) *p* = 0.059

The cumulative incidence of pleural recurrence or malignant pleural effusion was significantly higher in PLC3-positive patients than in PLC3-negative patients (*p* = 0.003; Fig. 3a). Stratification by the total number of PLC-positive results (single vs. double vs. triple; Fig. 3b) showed that triple-positive patients had a significantly higher cumulative incidence of pleural recurrence than single-positive patients (*p* = 0.046), whereas the double- vs. triple-positive comparison was not significant (*p* = 0.378). Further analysis based on the PLC1/PLC3 combinations defined three groups: (1) PLC3-negative (regardless of PLC1), (2) persistently positive (PLC1 + /PLC3 +), and (3) conversion-to-positive (PLC1–/PLC3 +). The persistently positive group had a significantly higher incidence of pleural recurrence than the PLC3-negative group (*p* = 0.0005) and showed a non-significant trend toward higher incidence relative to the conversion-to-positive group (*p* = 0.059; Fig. 3c).

The clinicopathological correlates are shown in Table 3. PLC3-positive patients had significantly higher rates of pathological lymph node metastasis (*p* = 0.003), lymph node dissection (*p* = 0.004), and LVI (*p* = 0.012) in comparison to PLC3-negative patients.

**Table 3:** Clinicopathological features of the PLC3-positive and PLC3-negative groups

Factors		PLC3 + *n* = 21	PLC3- *n* = 20	*p*-value
Pathological nodal involvement	Present/Absent	12 (57%)/9 (43%)	2 (10%)/18 (90%)	0.003
Lymph node dissection	Yes/No	18 (86%)/3 (14%)	8 (40%)/12 (60%)	0.004
Lymphovascular invasion	Present/Absent	16 (76%)/5 (24%)	7 (35%)/13 (65%)	0.012
Pathological pleural invasion	Present/Absent	13 (62%)/8 (38%)	7 (35%)/13 (65%)	0.121

Table 4 summarizes the Fine and Gray competing risks regression for RFS. In the univariate analysis, several factors were significantly associated with RFS. We then evaluated two scoring systems reflecting serial PLC results: (1) the number of positive PLC results (0–3) and (2) the combined PLC1/PLC3 pattern (PLC3-negative = 0, PLC1-negative/PLC3-positive = 1, PLC1-positive/PLC3-positive = 2). Both variables were strongly associated with RFS.

**Table 4:** Fine and Gray competing risks analysis for RFS

Variable	Univariate analysis		Multivariate analysis	
	Hazard ratio (95% CI)	p-value	Hazard ratio (95% CI)	*p*-value
Age	1.057 (1.019–1.096)	0.003		
Sex (Female = 0, Male = 1)	2.571 (1.534–4.309)	< 0.001		
Smoking (No = 0, Yes = 1)	2.169 (1.291–3.644)	0.004		
PLC1 (Negative = 0, Positive = 1)	3.459 (1.756–6.814)	< 0.001	1.751(0.827–3.710)	0.14
PLC3 (Negative = 0, Positive = 1)	4.687 (2.565–8.566)	< 0.001	3.056 (1.663–5.616)	< 0.001
Number of PLC positive results (0–3)	1.969(1.532–2.53)	< 0.001		
Combined PLC1/PLC3 score (0–2)	2.934(2.16–3.986)	< 0.001		
Surgical procedure (Sublobar = 0, Lobectomy/Pneumonectomy = 1)	1.060 (0.557–2.017)	0.860		
Lymph node dissection (No = 0, Yes = 1)	0.797 (0.490–1.300)	0.360		
Histology (AD = 0, non-AD = 1)	1.394 (0.814–2.386)	0.230		
Maximum Tumor Size (mm)	1.036 (1.024–1.047)	< 0.001		
Pathological nodal involvement (Negative = 0, Positive = 1)	2.416 (1.504–3.880)	< 0.001	1.318(0.757–2.295)	0.33
Lymphovascular invasion (Negative = 0, Positive = 1)	4.532 (2.733–7.516)	< 0.001	4.031(2.395–6.786)	< 0.001
Pathological pleural invasion (Negative = 0, Positive = 1)	2.160(1.326–3.519)	0.002		

*AD* adenocarcinoma

Combined PLC1/PLC3 score:

PLC1 + or PLC1-/PLC3- group = 0, PLC1-/PLC3 + group = 1, PLC1 + /PLC3 + group = 2

In the multivariate model, adjusted for non-lung cancer deaths, which included four covariates (PLC1 positivity, PLC3 positivity, LVI, and pathological nodal involvement), PLC3 positivity and LVI remained independent predictors of poor RFS (subdistribution hazard ratio [SHR] 3.056, 95% CI 1.663–5.616; *p* < 0.001 for PLC3; SHR 4.031, 95% CI 2.395–6.786; *p* < 0.001 for LVI), whereas PLC1 positivity (SHR 1.751, 95% CI 0.827–3.710; *p* = 0.14) and nodal status did not.

To further clarify the prognostic utility of scoring systems that incorporate serial PLC results (PLC1–3), we performed two additional multivariate analyses. First, a model that included the number of positive PLC results (0–3), maximum tumor size, LVI, and pathological nodal involvement demonstrated that the number of positive PLC results was an independent predictor of RFS (SHR 1.510, 95% CI, 1.133–2.014; *p* = 0.005; Supplementary Table 1). Second, when the combined PLC1/PLC3 pattern was analyzed together with maximum tumor size, LVI, and pathological nodal involvement, the PLC1/PLC3 score remained an independent predictor (SHR 1.64, 95% CI 1.12–2.41; *p* = 0.012; Supplementary Table 2).

## Discussion

PLC has long been recognized as a prognostic factor for resected NSCLC, although it is not included in the current TNM staging system. In most institutions, PLC is performed only immediately after thoracotomy (PLC1) [6, 7] and not at chest closure (PLC3), and the instilled saline volume varies widely from 20 to 1000 mL [6, 9, 10]. Previous studies have demonstrated the prognostic value of both PLC1 [6] and PLC3 [7] individually; however, no prior investigation has systematically assessed the serial changes in PLC across multiple intraoperative sampling points.

In our study, patients whose cytology was negative at PLC3 after lavage had outcomes equivalent to those of patients with consistently negative cytology. In contrast, patients who remained positive or became positive at PLC3 developed pleural dissemination [11] or malignant effusion and had a significantly poorer prognosis. Persistent positivity at chest closure may reflect not only preexisting tumor cell contamination but also intraoperative dissemination caused by surgical manipulation. Indeed, among the 14 patients who converted from PLC1-negative to PLC2-positive, 13 patients had undergone lymph node dissection, and many exhibited adverse pathological features such as nodal involvement, LVI, or PL. These findings suggest that extensive manipulation, particularly lymph node dissection, may facilitate tumor cell spillage, consistent with previous reports linking aggressive surgical techniques to pleural dissemination [12–14]. Technical modifications, such as en bloc lymphadenectomy, may therefore help reduce intraoperative tumor cell dissemination [15, 16].

Another explanation for our findings relates to the biological behavior of circulating tumor cells within the pleural cavity. Free-floating tumor cells may be effectively removed by a lavage [10], whereas cells adherent to the pleural surface are more resistant to clearance [17, 18]. In particular, tumor cell clusters demonstrate enhanced metastatic potential compared to single cells, driven by the increased expression of adhesion molecules such as integrins and selectins [19]. Experimental studies have shown that such clusters, especially when enriched with cancer stem cells, display stronger interactions with the microenvironment, increasing their ability to attach, implant, and propagate on pleural surfaces [20]. These mechanisms likely underlie the poor prognosis of patients with persistent PLC3 positivity.

Since 2017, our institution has employed distilled water lavage after lung resection to exploit its osmotic tumoricidal effects. Huguet et al. demonstrated that distilled water achieved 100% tumor cell death in 14 min in vitro and 32 min in vivo in a colorectal cancer model [21]. Previous reports, particularly on abdominal surgery, have demonstrated that distilled water lavage exerts cancer cell-killing effects via hypotonic stress-induced cytocidal mechanisms, and some studies have even reported improvements in survival outcomes [22–25]. In our study, among the 37 patients who were positive prior to distilled water lavage, 20 (54.1%) converted to negative at PLC3, and none of these patients subsequently developed pleural recurrence or malignant effusion. This finding supports the efficacy of distilled water irrigation in eliminating free-floating tumor cells and highlights its potential role in intraoperative prophylaxis against pleural dissemination.

Further stratification revealed that patients who were persistently positive for PLC1 through PLC3 had the worst outcomes, while those who converted from negative at PLC1 to positive at PLC3 fared somewhat better, although still worse than PLC3-negative cases. This suggests that the persistent tumor burden present before resection may reflect a biologically more aggressive disease than tumor cells disseminated secondarily during surgery. Conversely, patients whose cytology was negative at PLC3 had outcomes indistinguishable from those of consistently negative cases, underscoring the clinical importance of the final cytology status.

This study had several limitations. First, its retrospective, single-center design introduces the risk of selection bias and may limit its generalizability to other settings. Second, the relatively small number of events and limited sample size constrained the statistical power, precluding more granular subgroup analyses. Third, heterogeneity in lavage volume, timing, and cytological processing, as well as inter-observer variability, may have introduced measurement errors. Fourth, PLC has imperfect sensitivity and specificity, raising the possibility of misclassification. Finally, a median follow-up period of 24.5 months may be insufficient to capture very late recurrences, and external validation in larger multicenter cohorts is necessary.

## Conclusion

PLC3 positivity was strongly associated with pleural dissemination, malignant effusion, and poor RFS, whereas PLC1 positivity alone was insufficient to predict the outcomes. Patients who remained positive for PLC1 through PLC3 had the worst prognosis, while those who became PLC3-negative had outcomes equivalent to consistently negative cases. Although PLC2 positivity may reflect intraoperative tumor cell spillage, its clinical utility in prognostication is limited. These findings suggest that performing PLC at both thoracotomy (PLC1) and chest closure (PLC3) provides more accurate prognostic information than performing PLC1 alone. Incorporating PLC3 into routine clinical practice may improve risk stratification and guide decisions regarding adjuvant therapy.

## Data Availability

All data have been included in this article. Further inquiries can be directed to the corresponding author.
